# Supplementary figures and images for: Impact of fruit-tree shade intensity on the growth, yield, and quality of intercropped wheat
Source: PLoS One. 2019 Apr 2;14(4):e0203238. doi: 10.1371/journal.pone.0203238 (PMC6445427; doi:10.1371/journal.pone.0203238)

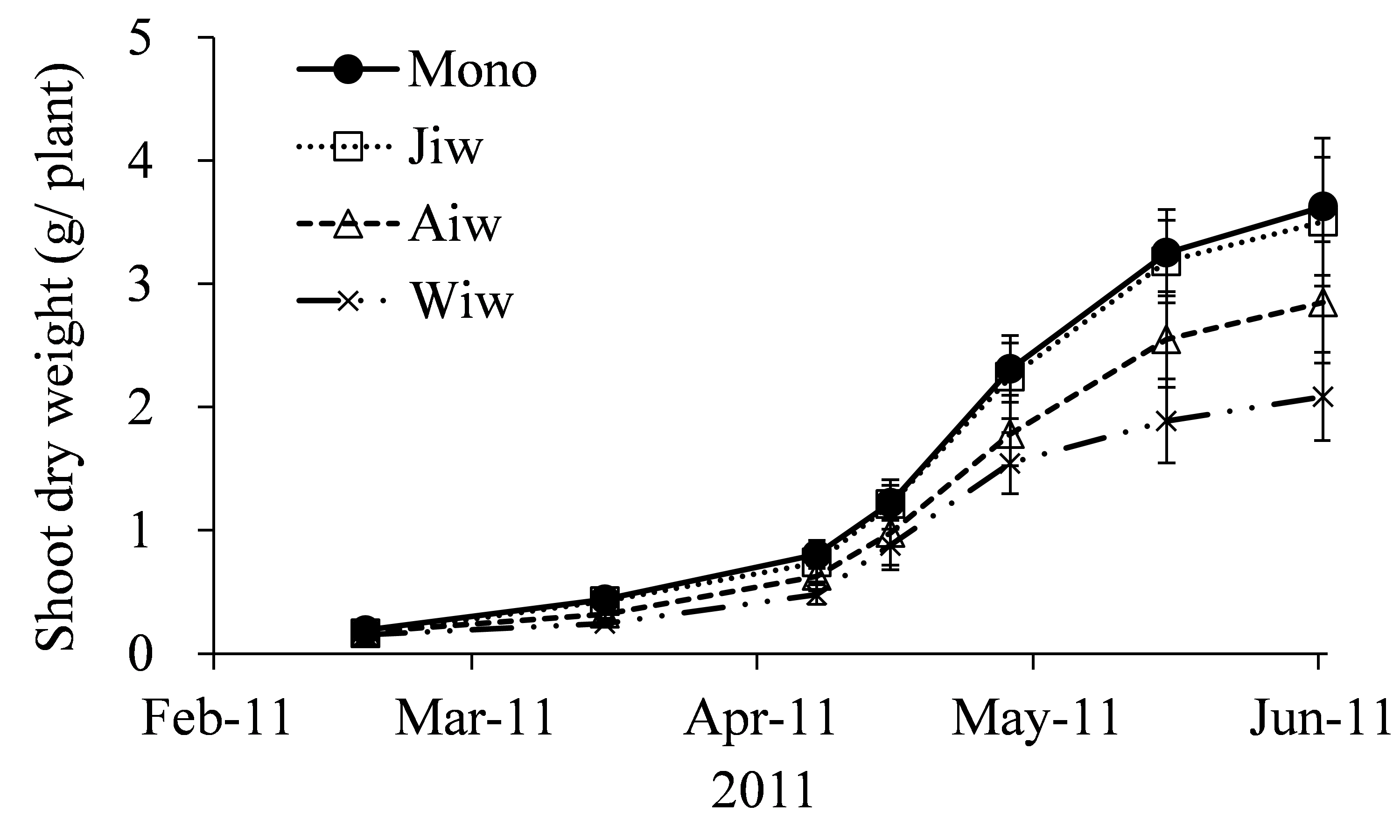

Supplement: S1 Fig — The data indicates the mean values of east, middle and west regions. Mono, monoculture wheat system; Jiw, jujube-wheat intercropping system; Aiw, apricot-wheat intercropping system; Wiw, walnut-wheat intercropping system. (TIF) [file pone.0203238.s001.tif]

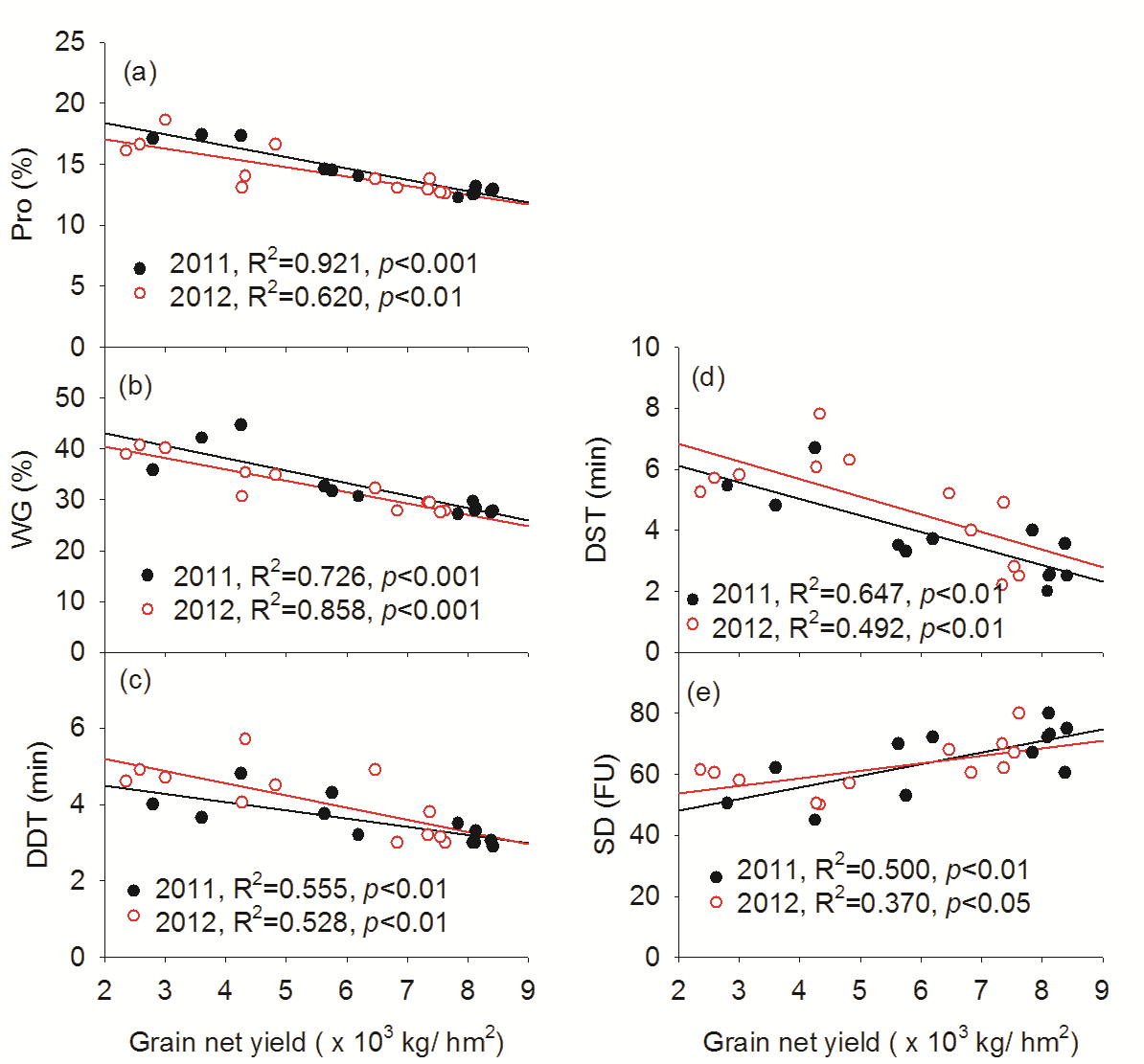

Supplement: S2 Fig — Note: Pro: Protein content; WG: Wet gluten content; DDT: Dough development time; DST: Dough stability time; SD: Softening degree. (TIF) [file pone.0203238.s002.tif]

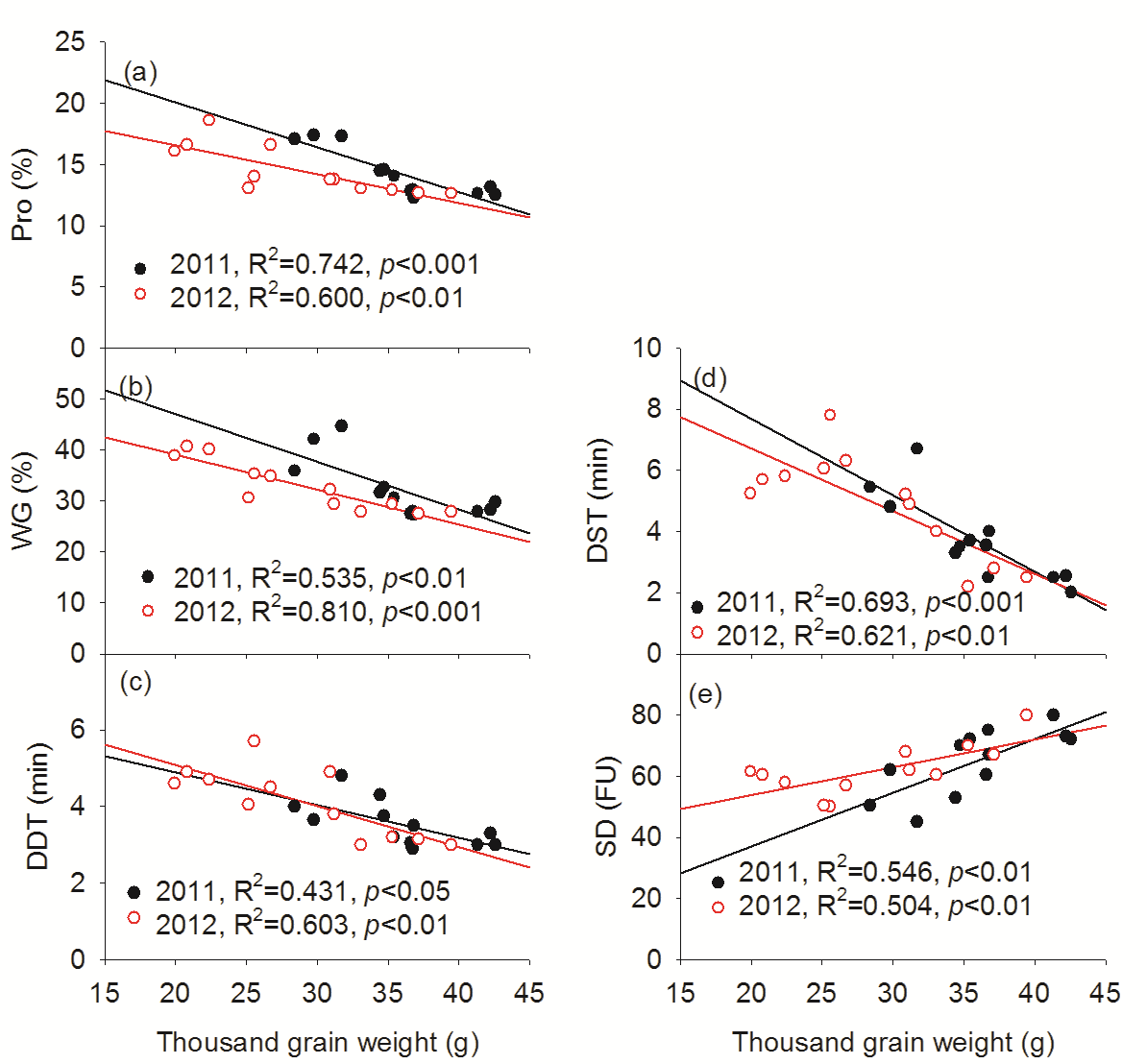

Supplement: S3 Fig — Pro: Protein content; WG: Wet gluten content; DDT: Dough development time; DST: Dough stability time; SD: Softening degree. (TIF) [file pone.0203238.s003.tif]
