# Supplementary material for: Impact of fruit-tree shade intensity on the growth, yield, and quality of intercropped wheat
Source: PLoS One. 2019 Apr 2;14(4):e0203238. doi: 10.1371/journal.pone.0203238 (PMC6445427; doi:10.1371/journal.pone.0203238)
Supplement: S1 Table — Note: Mono, monoculture wheat system; Jiw, Jujube-wheat intercropping system; Aiw, apricot-wheat intercropping system; Wiw, walnut-wheat intercropping system. (DOCX) [file pone.0203238.s004.docx]

**S1 Table.**

|  | pH | Organic matter | Total N | Total P | Total K | Available N | Olsen-P | NH_4_OAc-K |
| --- | --- | --- | --- | --- | --- | --- | --- | --- |
|  |  | (g kg^-1^) | (g kg^-1^) | (g kg^-1^) | (g kg^-1^) | (mg kg^-1^) | (mg kg^-1^) | (mg kg^-1^) |
| Mono | 8.75 | 8.08 | 0.69 | 1.21 | 19.1 | 67.2 | 30.3 | 91.8 |
| Jiw | 8.66 | 11.1 | 0.56 | 0.98 | 20.5 | 48.6 | 32.1 | 122.4 |
| Aiw | 8.71 | 10.42 | 0.63 | 1.02 | 18.3 | 55.3 | 26.5 | 113.7 |
| Wiw | 8.62 | 11.23 | 0.71 | 1.05 | 19.8 | 58.9 | 27.2 | 101.5 |
