# Supplementary material for: Impact of fruit-tree shade intensity on the growth, yield, and quality of intercropped wheat
Source: PLoS One. 2019 Apr 2;14(4):e0203238. doi: 10.1371/journal.pone.0203238 (PMC6445427; doi:10.1371/journal.pone.0203238)
Supplement: S2 Table — Note: Mono, monoculture wheat system; Jiw, jujube-wheat intercropping system; Aiw, apricot-wheat intercropping system; Wiw, walnut-wheat intercropping system. (DOCX) [file pone.0203238.s005.docx]

**S2 Table.**

| Year | Treatment | Stalks | | |  | Grains | | |
| --- | --- | --- | --- | --- | --- | --- | --- | --- |
|  |  | N (mg/g) | P (mg/g) | K (mg/g) |  | N (mg/g) | P (mg/g) | K (mg/g) |
| 2011 | Mono | 5.20±0.62b | 0.39±0.04b | 27.6±1.9b |  | 18.7±0.7b | 3.32±0.39b | 41.6±1.5c |
|  | Jiw | 5.67±0.55b | 0.37±0.05b | 27.7±2.8b |  | 18.2±2.4b | 3.20±0.36b | 41.5±0.5c |
|  | Aiw | 6.07±0.47b | 0.39±0.03b | 32.7±2.3a |  | 20.9±0.4ab | 3.63±0.25ab | 46.3±2.6b |
|  | Wiw | 8.47±1.52aa | 0.61±0.13a | 33.2±2.7a |  | 21.5±1.6a | 4.20±0.20a | 51.5±2.5a |
| 2012 | Mono | 6.1±1.6c | 0.32±0.01b | 28.7±1.3b |  | 17.9±0.5b | 3.54±0.01b | 46.0±3.4b |
|  | Jiw | 6.7±1.1bc | 0.37±0.01b | 28.3±0.8b |  | 18.5±0.6b | 3.54±0.22b | 45.9±5.5b |
|  | Aiw | 8.6±1.2ab | 0.36±0.02b | 29.1±1.5b |  | 23.1±1.5a | 4.02±0.36a | 56.4±3.1a |
|  | Wiw | 9.8±0.5a | 0.94±0.10a | 34.1±2.2a |  | 22.4±0.1a | 4.12±0.20a | 60.5±3.2a |
